# Association of early and later depressive symptoms with functional outcome after ischemic stroke

**DOI:** 10.1007/s00702-021-02328-w

**Published:** 2021-03-16

**Authors:** Anna Maria Lopatkiewicz, Joanna Pera, Agnieszka Slowik, Tomasz Dziedzic

**Affiliations:** Department of Neurology, Jagiellonian University Medical College, ul. Botaniczna 3, 31-503 Krakόw, Poland

**Keywords:** Stroke, Depression, Delirium, Outcome, Cognitive decline

## Abstract

**Background:**

Post-stroke depressive symptoms (DS) can be chronic or transient, occurring shortly or long after stroke and lasting only few months. It remains unclear if the prognosis differs between patients with DS in the acute phase of stroke and those who develop DS several months later. We aimed to determine whether outcomes vary among patients with different trajectories of post-stroke depressive symptoms.

**Methods:**

Of 698 enrolled patients with ischemic stroke, we included 335 participants (median age: 68, 48% female) who were assessed for DS both 8 days and 3 months post-stroke. We divided patients into 4 groups: without greater DS (Group 1), only earlier DS (Group 2), only later DS (Group 3), and persistent DS (Group 4). Logistic regression was used to determine the association between DS and 3- and 12-month functional outcome.

**Results:**

Group 2 was predominantly female and had the highest rate of previous stroke or transient ischemic attack. Group 3 was more likely to suffer from delirium and more severe stroke. Group 4 had the highest frequency of vascular risk factors, pre-morbid psychiatric symptoms, and cognitive decline. In multivariate analysis, Group 3, but not Groups 2 and 4, had an increased risk of poor 3- and 12-month functional outcome (adjusted OR 2.59, 95% CI 1.64–4.07, *P* < 0.01 and OR 3.97, 95% CI 2.32–6.76, *P* < 0.01, respectively) compared with Group 1.

**Conclusions:**

Different trajectories of post-stroke DS are related to different outcomes. Patients who only have later DS also have the worst prognosis.

## Introduction

The prevalence of depression after stroke is about 30% and remains stable up to 10 years, with a cumulative incidence of 39–52% within 5 years after stroke (Ayerbe et al. 2013; Hackett and Pickles 2014).

Post-stroke depression is associated with worse functional outcome (Ayerbe et al. 2013; Kutlubaev and Hackett 2014), increased mortality (Bartoli et al. 2013), a higher risk of recurrent stroke (Sibolt et al. 2013) and lower quality of life (Ayerbe et al. 2013).

Longitudinal studies that examined the natural history of post-stroke depression demonstrated a dynamic course of depressive symptoms, with new cases and recovery from depression occurring over time (Ayerbe et al. 2013). Thus, post-stroke depressive symptoms could be transient, occurring early or late after stroke onset and lasting only few months, or chronic. Assessment of post-stroke depressive symptoms at only a single time point might overlook patient heterogeneity and be unable to detect varying patterns of change in variables over time. Little is known about the relationship between time course of post-stroke depressive symptoms and outcome. We aimed to determine whether outcomes (3- and 12-month functional outcome, and 12-month case fatality) vary among patients with different trajectories of post-stroke depressive symptoms.

## Methods

### Patient selection and clinical assessment

We recruited patients to this study among participants of the PROPOLIS study (PRospective Observational POLIsh Study on post-stroke delirium). PROPOLIS was a prospective study conducted in the Department of Neurology, University Hospital, Krakow, Poland (Klimiec et al. 2015). The main goal of the PROPOLIS was to determine the frequency, risk factors and prognosis of post-stroke delirium. Participants were recruited to this study between May 2014 and March 2016.

The primary endpoint of this study was the 3-month functional outcome. The secondary endpoints were 12-month functional outcome and case fatality.

The inclusion criteria to the current sub-study on depressive symptoms were: (1) ischemic stroke or transient ischemic attack (TIA); (2) admission to the hospital within 48 h after stroke symptoms onset; (3) assessment of depressive symptoms in both time points (8 days and 3 months after stroke); and (4) informed patient’s consent. Patients for whom depressive symptoms could not be assessed due to severe aphasia, dementia, delirium or consciousness disturbances were excluded from the study.

The Bioethics Committee of Jagiellonian University approved the study’s protocol. Each patient gave informed consent.

We assessed the presence of depressive symptoms on day 8 ± 1 and 3 months after stroke onset using the Patient Health Questionnaire (PHQ-9) (Kroenke et al. 2001). Previous studies showed that PHQ-9 is a valid and clinically feasible depression screening tool for stroke (Burton and Tyson 2015). Score ≥ 10 was considered indicative of greater depressive symptoms (Williams et al. 2005; de Man-van Ginkel et al. 2013). Before the PHQ-9 was administrated, we examined aphasia using clinical methods to assess speech fluency and content, comprehension, and naming.

We divided patients into 4 groups. Group 1 (without depressive symptoms: D−/D −) consisted of patient who had no greater depressive symptoms both 8 days and 3 months after stroke. Group 2 (only earlier depressive symptoms: D+/D −) included patients who had greater depressive symptoms on day 8, but not 3 months after stroke. Group 3 (only later depressive symptoms: D−/D +) consisted of patients who had greater depressive symptoms 3 months after stroke but not on day 8. Patients in group 4 (persistent depressive symptoms: D+/D +) had both earlier and later depressive symptoms.

We used the Neuropsychiatric Inventory (NPI) to assess neuropsychiatric disturbances occurring within the 4 weeks before admission. The NPI-Q10 subscale includes ten behavioral items: delusions, hallucinations, agitation, depression, anxiety, euphoria, apathy, disinhibition, irritability, and aberrant motor behavior (Cummings 1997). A score for each item (from zero to 12) is a product of the severity scale (from zero to 3) and frequency scale (from zero to 4).

We used the Informant Questionnaire on Cognitive Decline in the Elderly (IQCODE) with a cut-off of 3.3 to diagnose pre-stroke cognitive decline (Jorm and Korten 1988; Harrison et al. 2016). The IQCODE consists of 26 items that rate change in patients’ intellectual abilities over the past 10 years.

We examined the core features of delirium daily during the first 7 days after admission using the Brief Confusion Assessment Method (Han et al. 2013). In addition, nurses completed daily questionnaire about patient’s behavior and cognitive fluctuations. We used the DSM-5 criteria for a diagnosis of delirium (American Psychiatric Association 2013).

We used the National Institute of Health Stroke Scale (NIHSS) to assess neurological deficit on admission (Brott et al. 1989) and the modified Rankin Scale (mRS) to assess functional outcome (van Swieten et al. 1988). Unfavorable outcomes were defined as a mRS of 3–6.

A trained psychologist or resident neurologist performed neuropsychiatric assessment. A senior neurologist with expertise in geriatric neurology adjusted the diagnosis of delirium. Functional outcome was assessed at 3 and 12 months after stroke in the outpatient clinic or by phone.

### Statistical analysis

We used the *χ*^2^ test to compare proportions and the Kruskal–Wallis test with Dunn’s post hoc test to compare continuous variables between groups. We considered *P* value less than 0.05 as significant.

We used logistic regression to determine the association between depressive symptoms and functional outcome. We adjusted all multivariate models for two of the most important predictors of stroke outcome: age and NIHSS score on admission. In the next step, in the multivariate models, we also included other variables that had *P* value below 0.05 in the univariate analysis. For calculations, we used the program STATISTICA for Windows (version 13.3, TIBCO Software Inc., Poland).

## Results

### Participants

**Fig. 1:**
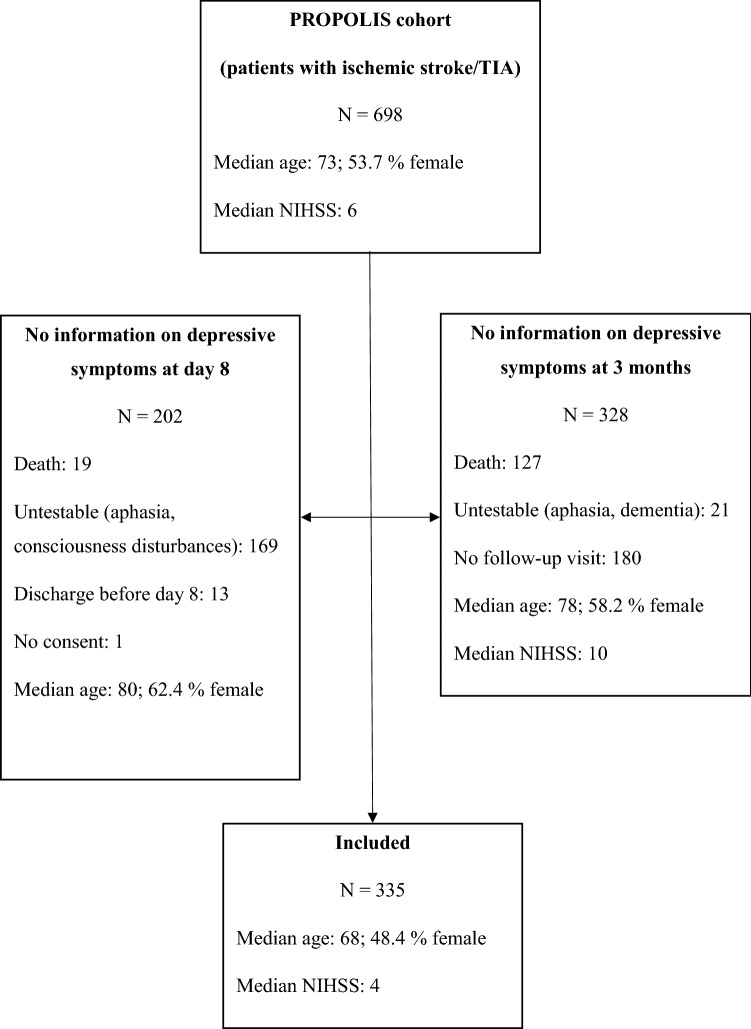
Flow chart showing the numbers of patients included in the study and the reasons for non-inclusion of excluded patients

Of 750 patients who participated in the PROPOLIS study, 698 patients had an ischemic stroke or TIA. Among these, 335 patients underwent depressive symptoms assessment both 8 days and 3 months after stroke (Fig. 1).

Tables 1 and 2 show the baseline characteristics and outcomes of the study groups.

**Table 1:** Baseline characteristics and outcomes of study groups

	Group 1 (*n* = 213) (D−/D** −**)	Group 2 (*n* = 41) (D+/D** −**)	Group 3 (*n* = 46) (D−/D +)	Group 4 (*n* = 35) (D+/D +)	*P* value
Age, median (IQs)	68 (60–78)	65 (58–79)	65.5 (59–81)	71 (67–76)	0.57 H(3) = 1.99
Female, *n* (%)	87 (40.8)	29 (70.7)	25 (54.3)	21 (60.0)	< 0.01
Ischemic stroke, *n* (%)	195 (91.5)	37 (90.2)	41 (89.1)	34 (97.1)	0.60
TIA, *n* (%)	18 (8.5)	4 (9.8)	5 (10.9)	1 (2.9)	
Risk factors and co-morbidities					
Hypertension, *n* (%)	139 (65.3)	27 (65.8)	36 (78.3)	30 (85.7)	0.04
Atrial fibrillation, *n* (%)	37 (17.4)	6 (14.6)	3 (6.5)	9 (25.7)	0.12
Diabetes mellitus, *n* (%)	50 (23.5)	9 (21.9)	11 (23.9)	16 (45.7)	0.04
Myocardial infarction, *n* (%)	28 (13.1)	6 (14.6)	7 (15.2)	6 (17.1)	0.92
Previous stroke/TIA, *n* (%)	31 (14.5)	13 (31.7)	8 (17.4)	9 (25.7)	0.04
Pre-stroke cognitive decline, *n* (%)*	18/181 (9.9)	3/37 (10.8)	7/38 (18.4)	6/27 (22.2)	0.19
NPI score, median (IQs)*	0 (0–6)	3 (0–7)	2 (0–9)	8 (1–16.5)	0.01# H(3) = 12.98
Anti-depressants prior stroke, *n* (%)	2 (0.9)	2 (4.9)	2 (4.3)	2 (5.7)	0.14
Stroke characteristics					
NIHSS score on admission, median (IQs)	4 (2–7)	3 (2–8)	5.5 (3–11)	3 (2–7)	0.03## H(3) = 9.58
Stroke location					0.29
Right hemisphere, *n* (%)	78 (36.6%)	13 (31.7)	21 (45.6)	21 (60.0)	
Left hemisphere, *n* (%)	103 (48.4)	21 (51.3)	17 (37.0)	12 (34.3)	
Posterior fossa, *n* (%)	30 (14.1)	6 (14.6)	7 (15.2)	2 (5.7)	
Multiple, *n* (%)	2 (0.9)	1 (2.4)	1 (2.2)	0 (0)	
Thrombolysis, *n* (%)	53 (24.9)	10 (24.4)	15 (32.6)	8 (22.9)	0.70
Mechanical thrombectomy, *n* (%)	10 (4.7)	3 (7.2)	3 (6.5)	3 (8.6)	0.75
Delirium, *n* (%)	20 (9.4)	7 (17.1)	14 (30.4)	4 (11.4)	< 0.01
Outcome					
Poor 3-month functional outcome, *n* (%)	32 (15.0)	10 (24.4)	24 (52.2)	10 (28.6)	< 0.01
Poor 12-month functional outcome, *n* (%)**	28/201 (13.9)	5/39 (12.8)	26/45 (57.8)	6/34 (17.6)	< 0.01
12-month case fatality, *n* (%)**	6/201 (3.0)	1/39 (2.6)	3/45 (6.7)	0/34 (0)	0.40
Use of anti-depressants during first 3 months after stroke, *n* (%)	17 (8.0)	8 (19.5)	12 (26.1)	4 (11.4)	< 0.01

*Data available for 283 patients

**Data available for 319 patients

^#^Post-hoc test: ***P = ***0.01 (group 1 vs group 4)

^##^Post-hoc test: ***P = ***0.02 (group 1 vs group 3)

**Table 2:** The summary of characteristic features of groups

	Group 1 (D−/D** −**)	Group 2 (D+/D** −**)	Group 3 (D−/D +)	Group 4 (D+/D +)
Female	+	+ +	+	+
Previous stroke/TIA	+	+ + +	+	+ +
≥ 2 vascular risk factors	+	+	+	+ +
Pre-stroke cognitive decline	+	+	+ +	+ +
Pre-stroke neuropsychiatric symptoms	+	+ +	+ +	+ + +
In-hospital delirium	+	+ +	+ + +	+
Stroke severity	+	+	+ +	+

The specific features of Group 2 (D+/D −) were female predominance (OR 2.92, 95% CI 1.43–5.97, *P* < 0.01) and the highest frequency of previous stroke or TIA (OR 2.38, 95% CI 1.15–4.93, *P* = 0.02) compared with other groups. The distinctive feature of Group 3 (D−/D +) was the highest rate of delirium (OR 3.64, 95% CI 1.75–7.58, *P* < 0.01) compared with other groups. This group had the highest NIHSS score on admission. Group 4 (D+/D +) had the highest rate of vascular risk factors (hypertension, diabetes mellitus, atrial fibrillation, and previous myocardial infarction). Compared with other groups, the patients from this group had at least 2 vascular risk factors more often (OR 3.91, 95% CI 1.81–8.46, *P* < 0.01). This group also had the highest NPI score. The rate of pre-stroke cognitive decline was the highest in Group 3 (D−/D +) and Group 4 (D+/D +).

### Primary endpoint

The outcomes of study groups are shown in Table 3.

**Table 3:** Results of uni- and multivariate logistic regression

	3-month poor functional outcome		12-month poor functional outcome	
	Univariate analysis	Multivariate analysis	Univariate analysis	Multivariate analysis
Group 2 (D+/D** −**) vs Group 1 (D−/D** −**)	OR 1.82 (95% CI 0.81–4.10), *P* = 0.14	OR 2.24 (95% CI 0.95–5.30), *P* = 0.06*	OR 0.91 (95% CI 0.32–2.53), *P* = 0.85	OR 0.94 (95% CI 0.31–2.80), *P* = 0.91*
		OR 1.97 (95% CI 0.76–5.12), *P* = 0.16**		OR 0.67 (95% CI 0.18–2.47), *P* = 0.54***
Group 3 (D−/D +) vs Group 1 (D−/D** −**)	OR 2.48 (95% CI 1.76–3.51), *P* < 0.01	OR 2.50 (95% CI 1.71–3.65), *P* < 0.01*	OR 2.91 (95% CI 2.03–4.16), *P* < 0.01	OR 3.20 (95% CI 2.12 – 4.83), *P* < 0.01*
		OR 2.59 (95% CI 1.64–4.07), *P* < 0.01**		OR 3.97 (95% CI 2.32–6.76), *P* < 0.01***
Group 4 (D+/D +) vs Group 1 (D−/D** −**)	OR 1.31 (95% CI 1.00–1.73), *P* = 0.05	OR 1.37 (95% CI 1.03–1.84), *P* = 0.03*	OR 1.10 (95% CI 0.79–1.52), *P* = 0.57	OR 1.08 (95% CI 0.77–1.53), *P* = 0.63*
		OR 1.24 (95% CI 0.87–1.76), *P* = 0.23**		OR 0.94 (95% CI 0.61–1.46), *P* = 0.81***

*Adjusted for age and NIHSS score

**Adjusted for age, NIHSS score, pre-stroke cognitive decline, NPI score, and delirium

***Adjusted for age, NIHSS score, hypertension, atrial fibrillation, pre-stroke cognitive decline, NPI score, and delirium

The predictors of 3-month poor functional outcome were: age (OR 1.04, 95% CI 1.02–1.06, *P* < 0.01), NIHSS score on admission (OR 1.13, 95% CI 1.08–1.19, *P* < 0.01), pre-stroke cognitive decline (OR 4.70, 95% CI 2.25–9.83, *P* < 0.01), NPI score (OR 1.03, 95% CI 1.00–1.06, *P* = 0.03), and delirium (OR 8.30, 95% CI 4.21–16.39, *P* < 0.01).

In the univariate analysis, Group 3 (D−/D +) and Group 4 (D+/D +) had an increased risk of poor functional outcome 3 months after stroke compared with Group 1 (D−/D −). In the fully adjusted model, Group 3 (D−/D +) had an increased risk of 3-month poor functional outcome compared with Group 1 (D−/D −). In the multivariate analysis adjusted for age and stroke severity, Group 4 (D+/D +) had an increased risk of poor 3-month outcome. After adjusting for other predictors, this risk was no longer significant.

Compared with all other groups, Group 3 (D−/D +) had higher risk of poor functional outcome (adjusted OR 5.42, 95% CI 2.32–12.66, *P* < 0.01).

### Secondary endpoints

The predictors of 12-month poor functional outcome were: age (OR 1.05, 95% CI 1.03–1.09, *P* < 0.01), NIHSS score on admission (OR 1.10, 95% CI 1.05–1.16, *P* < 0.01), hypertension (OR 2.22, 95% CI 1.12–4.39, *P* = 0.02), atrial fibrillation (OR 2.14, 95% CI 1.11–4.14, *P* = 0.02), pre-stroke cognitive decline (OR 3.22, 95% CI 1.46–7.08, *P* < 0.01), NPI score (OR 1.03, 95% CI 1.00–1.06, *P* = 0.04), and delirium (OR 6.71, 95% CI 3.31–13.58, *P* < 0.01).

Compared with Group 1 (D−/D −), Group 3 (D−/D +) had and increased risk of 12-month poor functional outcome in both uni- and multivariate analyses. Moreover, compared with all other groups, Group 3 (D−/D +) had higher risk of poor functional outcome 12 months after stroke (adjusted OR 13.58, 95% CI 5.31–43.72, *P* < 0.01).

In both uni- and multivariate analyses, the risk of poor 12-month outcome did not differ between Groups 1 and 2 as well as Group 1 and Group 4.

One-year case fatality did not differ between groups.

## Discussion

Our study revealed that a profile of predictors, associated factors and outcomes varies among patients with different courses of post-stroke depressive symptoms.

One of the features of Group 2 (D+/D −) was female predominance. Women have a roughly twofold higher risk of idiopathic major depressive disorder compared with men (Bromet et al. 2011). Women also appear to be more vulnerable to inflammation-induced depressive symptoms (Bekhbat and Neigh 2018; Lasselin et al. 2018). By contrast, two meta-analyses found no consistent association between sex and post-stroke depression (Ayerbe et al. 2013; Kutlubaev et al. 2014).

Patients from Group 2 (D+/D −) were also more likely to have history of stroke or TIA compared with other groups. Our previous study showed that a history of stroke is a predictor of early, but not late post-stroke depressive symptoms (Kowalska et al. 2020). In contrast, Eriksson et al. found an association between a history of stroke and self-reported depression 3 months after stroke (Eriksson et al. 2004).

The reduction in depressive symptoms in Group 2 (D+/D −) could reflect a natural resolution of post-stroke mood disturbances or could be a result of anti-depressive treatment. Transient depressive symptoms that occur shortly after stroke onset might be, at least in some patients, a part of the acute sickness response. Sickness behavior is a set of physiological and behavioral changes that occur during the course of infection and injury. It includes anorexia, fatigue, loss of interest, hyperalgesia, social withdrawal, sleep disturbances, and confusion (Dantzer et al. 2008). Mood disturbances, as part of an acute sickness response, are adaptive and short-lived. An acute sickness response model assumes that depressive symptoms occur around the time of stroke and remit spontaneously in most patients. One of the studies showed that the phenomenology of early-onset (in-hospital) post-stroke depression is different from the phenomenology of late-onset depression (Tateno et al. 2002). Patients with early-onset depression exhibited a higher frequency of vegetative symptoms, which may support the hypothesis that depressive symptoms that occur shortly after stroke onset are a component of an acute sickness response.

Overall, Group 2 (D+/D −) had a relatively good functional prognosis compared with other depressive groups. From a biological perspective, self-limited sickness behavior could be beneficial for a host (Harden et al. 2015). The persistence of mood disturbances beyond an acute phase of illness may suggest additional vulnerabilities (Maes et al. 2012).

One distinctive feature of group 3 (D−/D +) was the highest rate of delirium. Delirium is a serious acute neuropsychological disorder occurring in approximately 1 in 4 stroke patients (Shaw et al. 2019). It is associated with adverse outcomes, including increased mortality, functional decline and cognitive deterioration (Klimiec et al. 2016; Shi et al. 2012). Depression and delirium often co-exist and act as risk factors for each other (O'Sullivan et al. 2014). Several prospective studies of elderly patients after hip fracture or cardiac surgery found a positive association between delirium and subsequent depressive symptoms (O'Sullivan et al. 2014). In one of these studies, aged patients with hip fracture who had delirium were 1.5-times more likely to exhibit depressive symptoms at two-year follow-up (Dolan et al. 2000). Putative mechanisms linking delirium with depression might include alterations in monoamine transmission, and abnormal inflammatory and stress responses (O'Sullivan et al. 2014).

Additionally, compared with Group 1 (D−/D −) and Group 2 (D+/D −), Group 3 (D−/D +) was more likely to have pre-stroke cognitive decline which is a risk factor for both stroke-related delirium (Klimiec et al. 2016) and depression (Ayerbe et al. 2013; Kutlubaev et al. 2014).

Group 3 (D−/D +) had the worst functional outcome. The association between late depressive symptoms and prognosis remained independent of important prognosticators such as stroke severity, age, delirium and pre-stroke cognitive decline.

A specific feature of Group 4 (D+/D +) was the highest frequency of vascular risk factors and pre-morbid psychiatric symptoms. These patients also had a higher rate of pre-stroke cognitive decline. The frequency of poor functional outcome in this group was intermediate between Group 2 (D+/D −) and Group 3 (D−/D +).

Major cardiovascular risk factors such as hypertension appear to have no relation to post-stroke depression (De Ryck et al. 2014; Kutlubaev et al. 2014). An exception is diabetes mellitus, which was associated with post-stroke depressive symptoms in some studies (De Ryck et al. 2014; Kutlubaev et al. 2014). Clinically, Group 4 (D+/D +) shares some common features with so-called vascular depression which is characterized by the presence of vascular risk factors and cognitive deficits (Aizenstein et al. 2016; Taylor et al. 2013). The presence of cerebral vascular lesions on neuroimaging would be required to support the hypothetical link between persistent depressive symptoms after stroke with vascular depression.

Our study focused on depressive symptoms occurring in the acute and subacute phases of stroke. Ayis et al. analysed trajectories of depressive symptoms occurring in subacute and chronic phases of stroke (Ayis et al. 2016). They screened 761 patients for depressive symptoms at 3 months after stroke, and then annually up to 5 years. They identified four groups of patients. Group 1 (16%) included patients with no depressive symptoms. Patients in this group were more likely to be younger males with less severe stroke. Group 2 (49.5%) had patients with mild symptoms, and a tendency for a slight increase in symptoms over time. Group 3 (28.6%) represented patients with moderate symptoms, and a tendency for deterioration over time. Group 4 (6.4%) included patients with severe symptoms. This group had higher rates of impairments and female predominance.

Our study suggests a heterogeneity of post-stroke depressive symptoms. Further studies are needed to delineate different endophenotypes with putatively different biological underpinnings. A better understanding of the trajectories of depressive symptoms after stroke might be important for therapy. The EMOTION trial showed that patient responses to escitalopram depended on the presence of depression immediately following stroke. The efficacy of escitalopram was more marked in patients without depression at the time of randomization compared with those who had depression shortly after stroke onset (Lee et al. 2018). Patients who had depressive symptoms as a part of sickness behavior may not require anti-depressive treatment because these symptoms are usually self-limited.

Our study has several limitations. First, the most important consideration in interpreting our findings relates to representativeness of our cohort compared with the source population. Patients with severe stroke who die within 3 months after stroke onset and patients with severe aphasia or consciousness disturbances during the acute phase of stroke were excluded. As a result, our cohort included mostly patients with right-hemisphere lesions and milder stroke. Second, we did not use any psychiatric interviews to collect information about pre-morbid mood disorders. We have only gathered information about anti-depressants taken prior to stroke and we used the NPI, a validated informant-based interview, to assess neuropsychiatric symptoms over the previous month. Using the dichotomized scores of PHQ-9 as indicative of significant depressive symptoms, we could miss subtle changes in depressive symptoms. However, our approach reflects clinical routine where different questionnaires or scales with a validated cut-off point are used to screen depression. Additionally, some confounding factors related to depression, such as inflammation or obesity were not considered in our study (Sandu et al. 2015). Finally, a limited sample size and multiple comparisons are statistical limitations.

## Conclusions

Different trajectories of post-stroke depressive symptoms are related to different outcomes. Patients who only have later depressive symptoms also have the worst functional prognosis.

## Data Availability

The datasets used during the current study are available from the corresponding author on reasonable request.
